# Large Scale Identification of Osteosarcoma Pathogenic Genes by Multiple Extreme Learning Machine

**DOI:** 10.3389/fcell.2021.755511

**Published:** 2021-09-27

**Authors:** Zhipeng Zhao, Jijun Shi, Guang Zhao, Yanjun Gao, Zhigang Jiang, Fusheng Yuan

**Affiliations:** ^1^Department of Basic Medical Sciences, Taizhou University, Taizhou, China; ^2^Department of Orthopedics, Songyuan Central Hospital, Songyuan, China; ^3^Department of Orthopedics, The Fourth Affiliated Hospital of China Medical University, Shenyang, China; ^4^Department of Hand Surgery, Changchun Central Hospital, Changchun, China

**Keywords:** Index Term-osteosarcoma, pathogenic genes, fuses multiple extreme learning machine, machine learning, large scale identification

## Abstract

At present, the main treatment methods of osteosarcoma are chemotherapy and surgery. Its 5-year survival rate has not been significantly improved in the past decades. Osteosarcoma has extremely complex multigenomic heterogeneity and lacks universally applicable signal blocking targets. Osteosarcoma is often found in adolescents or children under the age of 20, so it is very important to explore its genetic pathogenic factors. We used known osteosarcoma-related genes and computer algorithms to find more osteosarcoma pathogenic genes, laying the foundation for the treatment of osteosarcoma immune microenvironment-related treatments, so as to carry out further explorations on these genes. It is a traditional method to identify osteosarcoma related genes by collecting clinical samples, measuring gene expressions by RNA-seq technology and comparing differentially expressed gene. The high cost and time consumption make it difficult to carry out research on a large scale. In this paper, we developed a novel method “RELM” which fuses multiple extreme learning machines (ELM) to identify osteosarcoma pathogenic genes. The AUC and AUPR of RELM are 0.91 and 0.88, respectively, in 10-cross validation, which illustrates the reliability of RELM.

## Introduction

Osteosarcoma is the most common malignant bone tumor in clinic (Marko et al., 2016), which is mostly seen in children and adolescents. Although surgery combined with neoadjuvant chemotherapy significantly improves the 5-year survival rate of patients with local tumors (Yang et al., 2018), most patients with osteosarcoma will metastasize, and the 5-year survival rate of patients with metastatic osteosarcoma is only 20 ∼ 30% (Murakami et al., 2017). At present, osteosarcoma is still the second leading cause of cancer-related death in adolescents (Chen et al., 2020). Considering the complex intra - and inter tumor heterogeneity, a suitable specific target for osteosarcoma has not been found. However, based on previous studies on the heterogeneity of other tumors, the immune microenvironment may have relatively low heterogeneity and become a more appropriate direction of intervention (Koirala et al., 2016). Therefore, identifying genes related to osteosarcoma immune microenvironment may provide a robust and effective target for clinical application (Mirabello et al., 2020).

According to the age at which osteosarcoma occurs suddenly increases with the onset of puberty, and its largest growth site is shown to be related to the rapid proliferation of bones, it indicates that osteosarcoma is significantly related to the rapid growth of bones (Ho et al., 2017). At the same time, exposure to alkylating agents may also promote the development of osteosarcoma (Zhang et al., 2021). In addition, radiotherapy is one of the few identified environmental risk factors for osteosarcoma. Studies have shown that increasing the radiation dose of primary cancer is linearly related to the risk of secondary osteosarcoma. Another study based on American adults also found that radiotherapy is significantly associated with an increased risk of osteosarcoma diagnosis in the future (Wu et al., 2012).

Whole-exome and whole-genome sequencing analysis of the germline DNA of patients with osteosarcoma showed that the prevalence of pathogenic variants in genes associated with known cancer susceptibility syndromes was higher than expected (Gianferante et al., 2017). Chromosomal abnormalities, pathogenic variants of tumor suppressor genes, transcription factors and growth factors, and abnormalities of WWOX and miRNA all play an important role in the occurrence and development of osteosarcoma (Lin et al., 2017). The frequency of osteosarcoma in individuals with mutations in the RB1 gene is higher than that in the population. Studies have shown that there is an interaction between primary inheritance and genes in the pathogenesis of the disease (Spritz, 2007). A 2016 study found that among individuals with pathogenic mutations in the germline tumor suppressor gene TP53, the cumulative incidence of osteosarcoma reached 5–11% (Mai et al., 2016). Transforming growth factor β (TGF-β) protein affects cell growth and metabolism, and the expression of TGF-β1 is significantly increased in highly malignant osteosarcoma. Insulin growth factors IGF-I and IGF-II can bind to the corresponding receptors to play a role, and they are overexpressed in osteosarcoma. The overexpression of CCN3 in osteosarcoma is related to its poor prognosis. Parathyroid hormone (PTH), parathyroid hormone related peptide (PTHrP) and parathyroid hormone receptor (PTHR1) have been shown to be related to the progression and metastasis of osteosarcoma (Berdiaki et al., 2010). Various molecular changes and genomes closely related to the occurrence and progress of osteosarcoma have been identified. These changes include gene amplification, deletion and germline mutation, overexpression and RTK activation, abnormal cell proliferation, metastasis, apoptosis, drug tolerance genes and miRNAs (Saraf et al., 2018). Osteosarcoma is characterized by complex and unbalanced karyotypes and abnormal gene expression profiles. Abnormalities of chromosome structure and value can be detected in most osteosarcoma (Isakoff et al., 2015). Common chromosome numerical abnormalities include germline mutation, deletion, polyploidy, aneuploidy, duplication and unbalanced ectopic errors (Morrow and Khanna, 2015). TP53 tumor gene and retinoblastoma tumor suppressor gene RB1 are the most prominent genes of germline mutation (Oliveira et al., 2005). They are the key detection sites of mitosis and the root cause of chromosome instability. Most osteosarcoma contains inactivation of both p53 and Rb pathways (Levine and Fleischli, 2000). In essence, the main causes of osteosarcoma are the inactivation of tumor suppressor gene expression and the abnormal doubling of oncogenes (Orr and Compton, 2013). Common oncogenes, such as avian cell homolog Myc, purine / pyrimidine exonuclease 1 (APEX1), action associated vascular endothelial growth factor A (VEGFA) and RecQ protein analog 4 (RecQL4). These amplified genes are closely related to the biological processes of osteosarcoma cell proliferation, growth and angiogenesis. Liu et al. (2019) identified 125 genes which are related to osteosarcoma and can be used to predict survival of osteosarcoma. Deng et al. (2021) used univariate, Lasso, and machine learning algorithm-iterative Lasso Cox regression analyses to predict survival of osteosarcoma by lncRNAs.

At present, there are two common biological methods for discovering disease-related genes. First, collect disease samples and health samples, respectively, conduct RNA-seq sequencing to obtain the expression of genes in different health states, and then obtain the genes significantly differentially expressed in disease and health populations through differential expression analysis (Zhao et al., 2021b). Second, through genome-wide association analysis, collect a large number of disease and healthy people, sequence the whole genome, and then compare the sequences to obtain sites with significant differences in mutation frequency (Peng and Zhao, 2020; Zhao et al., 2020c). However, both of them need a large number of samples to support in order to ensure the accuracy, which results in a large consumption of time and money (Bhakta and Tsukahara, 2020). With the continuous accumulation of biological data and the continuous improvement of calculation methods, bioinformatics experts find biological laws through calculation methods, and then infer more biological conclusions (Chen et al., 2019; Liu et al., 2020; Zhao et al., 2020a). The calculation methods have identified disease-related genes and drugs on a large scale (Tianyi et al., 2020; Zhao et al., 2020b). Although some conclusions are not completely accurate, they greatly reduce the scope of research and save time and money (Wu et al., 2021). Moreover, the models constructed by deep learning and machine learning can be used for reference by other research problems (Zhao et al., 2021a). Therefore, we developed a machine learning method to identify osteosarcoma-related genes in this paper. Using the idea of random forest for reference, we fused multiple Extreme Learning Machines (ELM) to build a model through the known osteosarcoma related genes to predict more genes potentially associated with osteosarcoma.

## Materials and Methods

### Workflow

**FIGURE 1:**
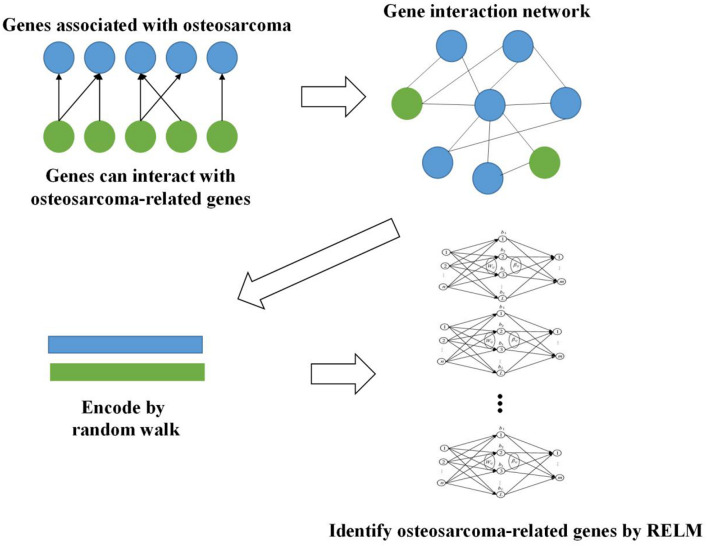
Workflow of our method.

Firstly, we obtained 2,339 genes which are reported to be related to osteosarcoma in DisGeNET (Piñero et al., 2020). Then, we constructed gene interaction network based on these genes. More genes are included in this network since many genes can interact with these 2,339 genes. We extracted the features of this network by random walk and used Random Extreme Learning Machine (RELM) to identify osteosarcoma-related genes. The way of constructing RELM is to build multiple ELM models and the output of each model is attached with weight, and the final result is obtained by voting. The whole workflow is shown in Figure 1.

### Extreme Learning Machine

The calculation process of single hidden layer neural network is as follows:

1. The input value is multiplied by the weight value
2. Add bias value
3. Calculation of activation function
4. Repeat steps 1 to 3 for each layer
5. Calculate output value
6. Error back propagation
7. Repeat steps 1 to 6.

Extreme learning machines improves it by removing step 4 and replacing step 6 with a primary matrix inverse operation and removing step 7.

The process of ELM is to construct the formula (1):


(1) $$f_{L}(x)=\sum_{i=1}^{L}\beta_{i}g_{i}(x)=\sum_{i=1}^{L}\beta_{i}g(w_{i}*x_{j}+b_{j}),j=1,\text{…},N$$

L is the number of hidden units. N is the number of training samples. *β*_*i*_ is the weight between *i*th hidden layer and output. *w*_*i*_ is the weight between input and output. *g*(*x*) is activation function. b is bias and x is the input. Since ELM only has one hidden layer, i is 1 in our model.

The calculation process of the extreme learning machine is very similar to the standard back-propagation neural network, but the weight matrix between the hidden layer and the output is a pseudo-inverse matrix. The above formula can be abbreviated as:


(2) T=Hβ

(3) $$H={\left[\begin{array}{c} g\left(w_{1}*x_{1}+b_{1}\right)\ldots g\left(w_{L}*x_{L}+b_{L}\right)\\ \vdots \;\vdots \\ g\left(w_{1}*x_{N}+b_{1}\right)\ldots g\left(w_{L}*x_{N}+b_{L}\right) \end{array}\right]}_{N\times L}$$

m is the number of outputs; H is the hidden layer output matrix; T is the target matrix of the training set.

### Random Extreme Learning Machine (RELM)

Extreme learning machines is a special artificial neural network with only one hidden layer, which causes its accuracy to be low. However, the calculation speed of ELM is extremely fast. Therefore, we can use this advantage to build multiple ELM models and use weighted voting to improve accuracy.

Random extreme learning machine draws on the idea of random forest (RF), regards ELM as a simple decision tree, and trains multiple ELMs to form an ELM forest to achieve the goal of improving accuracy.

The idea of RELM is to randomly extract the multi-dimensional features of genes, and then randomly extract the training set to form a simple ELM. Through repeated extraction with replacement, new ELMs are continuously trained. After getting enough ELM models, the final result is obtained by weighting and averaging the output results of the 500 models.

The number of features for each ELM model is selected as (Zhao et al., 2017):


(4) $$n=\sqrt{N}$$

N is the whole dimension of features. n is the number of features for each ELM model.

In the meanwhile, we randomly selected samples for each ELM model too. After each modeling, we will also put the sample back. We choose one-tenth of the samples for modeling each time.

## Results

### Selection of Extreme Learning Machine Model Number

We should construct multiple ELM models to obtain RELM, but the number of ELM models is not sure. Therefore, we tried 10, 20, 50, 100, 200 ELM models and used 10-cross validation to obtain the final number.

**FIGURE 2:**
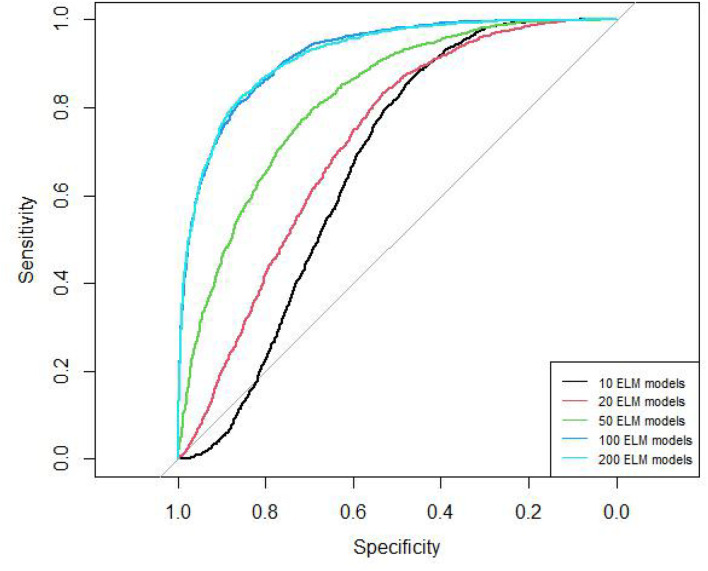
ROC curves of different ELM model number.

The AUC curves of 10, 20, 50, 100, 200 ELM models are shown in Figure 2. The AUC values of these models are 0.66, 0.72, 0.82, 0.92, 0.92, respectively. The AUC of 100 models and 200 models are similar.

**FIGURE 3:**
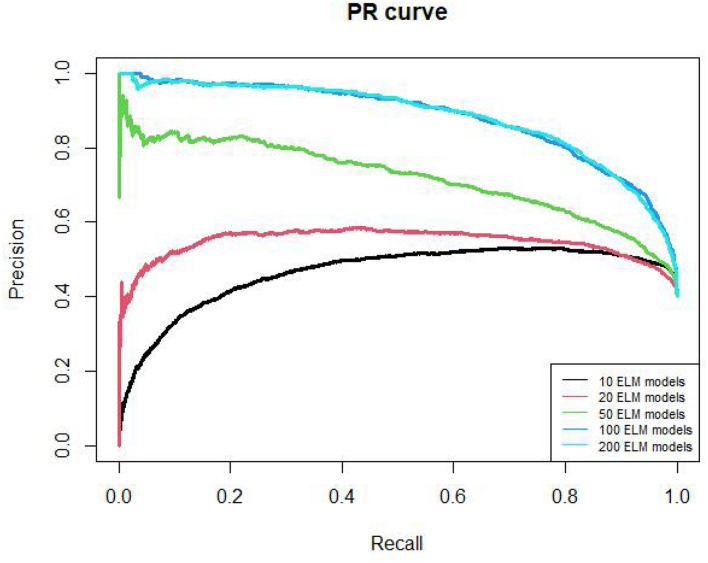
PR curves of different ELM model number.

The PR curves of 10, 20, 50, 100, 200 ELM models are shown in Figure 3. The AUPR values of these models are 0.46, 0.54, 0.72, 0.88, 0.88, respectively. The AUPR of 100 models and 200 models are similar too. Therefore, we chose 100 ELM models to construct RELM.

### Performance of Random Extreme Learning Machine

**FIGURE 4:**
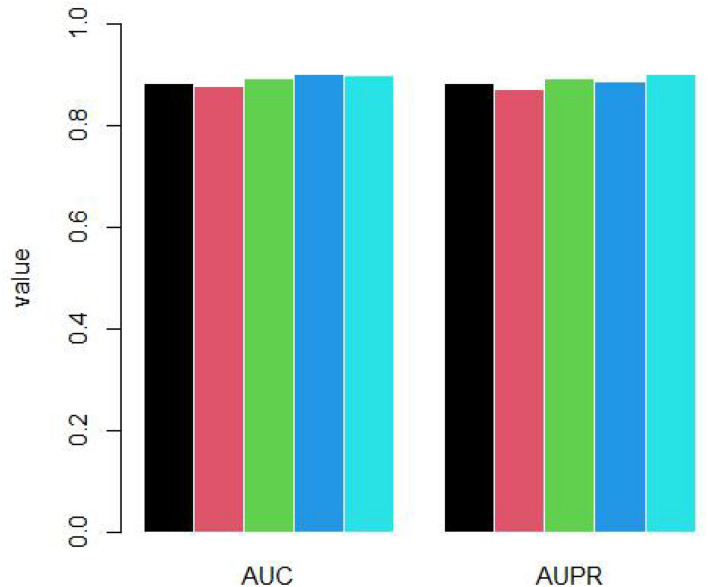
Five times 10-cross validation by randomly sampling.

Because the unknown genes are far more than known osteosarcoma-related genes, we randomly selected negative samples to build RELM model. For each time, the number of negative samples is as same as positive samples. We repeated to select negative samples 5 times and did 10-cross validation for each time. The AUC and AUPR is shown as Figure 4.

The mean AUC is 0.889 and standard deviation is 0.009. The mean AUPR is 0.887 and standard deviation is 0.011.

In order to further explore the advantages of RELM, we compared RELM with ELM, RSVM, RANN. RSVM is to replace the ELM of RELM by SVM and RANN is to replace ELM of RELM with ANN. The experiments results are shown in Table 1.

**TABLE 1:** Comparison result.

Method	AUC	AUPR
RELM	0.889	0.887
ELM	0.761	0.684
RSVM	0.865	0.841
RANN	0.876	0.849

As we can see in Table 1, RELM performed best among these method. SVM is more suitable for small sample modeling and ANN needs large sample set to build a precise model. Therefore, these two methods are not suitable for our case.

## Conclusion

Whole-exome and whole-genome sequencing analysis of the germline DNA of patients with osteosarcoma showed that the prevalence of pathogenic variants in genes associated with known cancer susceptibility syndromes was higher than expected. Osteosarcoma is highly aggressive and progresses rapidly. In all age groups, as many as 25% of patients have metastasized at the time of diagnosis, so its early diagnosis is necessary for the long-term prognosis of patients. At present, the diagnosis of osteosarcoma is still based on the patient’s clinical manifestations, imaging examinations and biopsy. Gene therapy includes tumor suppressor gene therapy, antisense gene therapy, suicide gene therapy and combined gene therapy. Although the research of gene therapy has made great progress and it has good therapeutic prospects, the clinical application of gene therapy still has a long way to go. In recent years, with continuous research on the key genes of osteosarcoma, its application value as a gene therapy target has gradually revealed.

To identify osteosarcoma-related genes in large scale, in this paper, we developed an ELM-based method for identifying osteosarcoma-related genes. 100 ELM models have been constructed to build a final RELM model. By constantly randomly selecting negative sets, we performed five times of 10-cross validation. The accuracy of RELM is stable and high in all experiments.

Overall, we purposed a reliable method for identifying osteosarcoma-related genes in large-scale. This method could help understand the pathogenesis of osteosarcoma and develop drug targets.

## Data Availability Statement

The datasets presented in this study can be found in online repositories. The names of the repository/repositories and accession number(s) can be found below: gene-disease associations: https://www.disgenet.org/ and gene interaction: http://www.inetbio.org/humannet.

## Ethics Statement

Ethical review and approval was not required for the study on human participants in accordance with the local legislation and institutional requirements. Written informed consent for participation was not required for this study in accordance with the national legislation and the institutional requirements.

## Author Contributions

ZZ, JS, and FY conceived and designed this study. ZZ, JS, GZ, YG, and ZJ analyzed the data. ZZ, JS, and GZ wrote the manuscript. All authors read and approved the final version of the manuscript.

## Conflict of Interest

The authors declare that the research was conducted in the absence of any commercial or financial relationships that could be construed as a potential conflict of interest.

## Publisher’s Note

All claims expressed in this article are solely those of the authors and do not necessarily represent those of their affiliated organizations, or those of the publisher, the editors and the reviewers. Any product that may be evaluated in this article, or claim that may be made by its manufacturer, is not guaranteed or endorsed by the publisher.
